# Mental health survey of medical personnel during pre-job training in a closed-loop management system during the COVID-19 pandemic

**DOI:** 10.3389/fpubh.2023.1279153

**Published:** 2023-11-08

**Authors:** Limin Zhou, Ximei Huang, Liping He, Jiaxin He, Jue Qin, Zhenling Fang, Chen Huang, Jinyu Chen

**Affiliations:** Guangzhou Panyu Central Hospital, Guangzhou, China

**Keywords:** pre-job training, closed-loop work system, mental health, medical personnel, COVID-19

## Abstract

**Object:**

With the aim of enhancing prevention and regional control of epidemics, the mental health status of medical personnel was analyzed before the implementation of closed-loop management during the COVID-19 pandemic in the regional hospital representative.

**Methods:**

In accordance with directives from the unified deployment of the national and regional health bureaus, and following the inclusion and exclusion criteria, from September 2021 to November 2022, all medical personnel assigned to a closed-loop working environment by Guangzhou Panyu Central Hospital were enrolled as research subjects through cluster sampling method. Using a cross-sectional survey method, relevant data such as age, gender, professional title, and mental health status were collected. The Patient Health Questionnaire-9 (PHQ-9) scale, the Generalized Anxiety Disorder-7 (GAD-7) scale, and the Insomnia Severity Index (ISI) scale were administered. Logistic regression analysis was used to study the influencing factors of depression, anxiety, and insomnia. Single factor logistic regression analysis was performed first, followed by multiple factor logistic regression analysis.

**Results:**

A total of 500 valid responses were received. Depression was reported by a higher proportion of physicians than nurses. Anxiety was reported by higher proportion of men than women and by a higher proportion of physicians than nurses. Medical personnel under the age of 30 years reported fewer symptoms of insomnia than those over the age of 41 years, and medical personnel with intermediate professional titles reported more severe symptoms of insomnia than junior personnel. There was no significant difference between the results of the three questionnaires for medical personnel from other hospital departments or in the different type of closed-loop work environments.

**Conclusion:**

During the pandemic, conducting psychological health assessments for medical personnel undergoing pre-job training in closed-loop management was beneficial for the timely detection of psychological problems. Although this study only conducted a cluster sampling survey and lacked comparative analysis on other medical institutions, it still suggested that it was necessary to strengthen timely psychological counseling and intervention for senior male physicians.

## 1. Introduction

In January 2020, Guangzhou Panyu Central Hospital became a designated hospital for the treatment of suspected cases of COVID-19 in our region. For prevention and control in an epidemic, it is important to contain and control the infection, disrupt the transmission route, and protect vulnerable population (1), so in March 2020, China implemented a prevention and control policy of centralized isolation for medical observation. The following month, our hospital dispatched medical personnel to a hotel to conduct medical observation of inbound personnel. In November of the same year, our hospital took over the medical operations of isolation hotels, the risk of overseas imports also persists (2, 3).

Subsequently, in September 2021, in accordance with notification documents such as the “Notice of the COVID-19 Prevention and Control Notification Office of Guangzhou City on Further Strengthening the Hierarchical Management of Workers in Centralized Isolation Sites,” we worked to strengthen the management of personnel dispatched to centralized isolation sites, to ensure that the work conducted there was standardized and stable and to establish a multi-dimensional prevention and control management system. To this end, our hospital formulated management regulations for medical personnel dispatched to work in a closed-loop management system (including those at isolation hotels), introduced special shifts for negative pressure transfer, and established a negative pressure isolation ward among other measures.

Closed-loop management refers to the unified implementation of a closed-loop management system for personnel in high-risk and low-risk positions. Management of personnel in high-risk positions included deciding a fixed number of staff and positions, arranging a centralized residence for them, and ensuring they were fully vaccinated. Management of personnel in low-risk positions included assigning them and making sure they were fully vaccinated. Also, in accordance with the “higher than the social level” prevention and control standard, the “two points, one line” policy for commuting from home to work or other isolation sites was implemented.

Closed-loop management personnel were required to strictly implement sign-in and sign-out procedures in the workplace as well as arrange a centralized residence along with shuttle services. Special shift staff for negative pressure transfer were required to keep attendance and departure records. Personnel were not to gather or socialize at the entrance and to use appropriate personal protective equipment (4). Before entering the closed loop, personnel were required to participate in pre-job training, which was organized by various departments, including the medical department, hospital infection management department, and human resources department.

In November 2022, our hospital set up two shelter hospitals, one top-level transfer station, and one health monitoring point, and high-risk and low-risk positions in the closed-loop management system were decided. Heavy workloads and the reality of work without a social life (5) is known to lead to different levels of depression, anxiety, and insomnia among medical personnel (6). This study involved a retrospective analysis of the mental health of medical personnel during pre-job training for closed-loop management, and analyzed various characteristics of the medical personnel, including age and gender. Specifically, we investigated factors associated with anxiety, depression, and insomnia. The results should enable hospitals to implement appropriate intervention measures after medical personnel enter the closed-loop work environment, including organizing care, training, and stress-relieving activities, providing psychological counseling, and enhancing salary and welfare benefits.

The results of this research should enable hospitals to help their medical staff by providing humanistic care, reducing work stress, and improving work satisfaction. The findings also provide a reference for managing the physical and mental health of the medical staff who function as the main health care providers when public health emergencies occur.

## 2. Methods

### 2.1. Participants

This study was approved by the medical ethics committee of Guangzhou Panyu Central Hospital (Reference number: PYRC-2023-080). Informed consent was obtained from every medical staff prior to participation in the study.

Inclusion criteria: (1) Age: 20–60 years old, with an independent civil capacity. (2) Health care worker working in a medical institution, including doctors, nurses, technicians, etc. (3) Physically healthy and willing to participate in psychological research.

Exclusion criteria: (1) Individuals under 20 years and over 60 years of age. (2) Not satisfying professional requirements, such as not having a practicing certificate or relevant technical title. (3) Having a history of mental illness or related family history. (4) Having received psychological counseling or other psychotherapy within the past 6 months. (5) Unwilling to cooperate with research requirements, such as refusing to answer questionnaires or participate in relevant tests. (6) Severe cardiovascular and cerebrovascular diseases, chronic heart failure, liver failure, renal failure, etc.

Between September 2021 to November 2022, medical personnel according with inclusion criteria were required to undergo pre-job training before being dispatched by the hospital to a closed-loop work environment, including the isolation hotels, negative pressure ward, support and control area, transfer station, and shelter hospital.

### 2.2. Research method

The cross-sectional survey method was applied. Before entering the closed-loop worksites, the participants received pre-job training and were asked to complete a questionnaire survey comprising the Patient Health Questionnaire (PHQ-9) (7–9), the Generalized Anxiety Disorder (GAD-7) (7–9), and the Insomnia Severity Index (ISI) (8, 10). In addition, the characteristics of the participants (e.g., age, discipline, and type of closed-loop work) were statistically analyzed. This study was the structured and standardized questionnaires. The Patient Health Questionnaire (PHQ-9) consists of 9 items, with a 4-point scoring system ranging from 0 to 3 points, for a total possible score of 27 points. The higher the score, the more frequent and severe the occurrence of depressive symptoms. The scores of 5, 10, 15, and 20, respectively, represent the boundary values for mild, moderate, moderate, and severe depression (9). The Generalized Anxiety Disorder (GAD-7) consists of 7 items, with a 4-point scoring system ranging from 0 to 3 points, for a total possible score of 21 points. The higher the score, the more severe the anxiety symptoms. A score of 5–9 indicates mild anxiety, a score of 10–14 indicates moderate anxiety, and a score of 15–21 indicates severe anxiety (7). The Insomnia Severity Index (ISI) scale consists of 7 items, with a 5-point scoring system ranging from 0 to 4 points, for a total possible score of 28 points. The higher the score, the greater the severity of the insomnia. A score of 0–7 indicates insomnia without significant clinical manifestations; 8–14 points is classified as mild insomnia; 15–21 points is classified as moderate insomnia; and 22–28 points is classified as severe insomnia (10).

### 2.3. Questionnaire settings

The psychological survey questionnaire was reliable and valid. The investigative procedure strictly adhered to the principles of ethics and privacy. The questionnaire contained cover of survey, title, date and filling instructions. In addition, basic information included the following items: age, gender, personnel category, title, etc. Three questionnaires, respectively, asked participants about the frequency and extent of mental health problems, anxiety problems, and insomnia in the past 2 weeks.

### 2.4. Data collection

Initially, participants completed the three written questionnaires during unified pre-job training. The data collection method was later changed: after professional training was completed by each department, all participants completed an online Jiandaoyun questionnaire survey before entering a closed-loop work environment and filed it as working data.

### 2.5. Statistical methods

Data are expressed as the mean ± standard deviation. The survey results are expressed as numbers and percentages, and the χ^2^ distribution formula was used for testing. Logistic regression analysis was performed to identify the factors associated with depression, anxiety, and insomnia self-reported by the participants. Univariate logistic regression analysis was performed first, but because few factors were identified as significant, all factors were entered into multivariate logistic regression analysis. Forward stepwise logistic regression was used to screen the variables (P_in_ = 0.05, P_out_ = 0.10). The odds ratio (OR) and 95% confidence interval were calculated for each significant factor. Statistical significance was established at *p* < 0.05. Intergroup differences were compared using the χ^2^-test, with the level of significance adjusted for the number of groups as follows: α′ = α/k, where k = m(m−1)/2 and m is the number of groups. All statistical analysis was performed using SPSS software ver. 26.0 (IBM, Armonk, NY).

## 3. Results

Among the 500 respondents prior to entering a closed-loop working environment, 246 (49.2%) were under 30 years of age, 195 (39%) were between 31 and 40 years, and 59 (11.8%) were over 41 years of age; 123 were men (24.6%) and 377 were women (75.4%). Nursing was the most represented discipline (*n* = 313 [62.6%]). Most participants had junior professional titles (*n* = 346 [69.2%]). Closed-loop workers comprised 226 personnel (45.2%) in the support and control area, 138 (27.6%) in the shelter hospital or transfer station, and 136 (27.2%) in the negative pressure ward or isolation hotels.

### 3.1. Prevalence of self-reported depression

The results of the PHQ-9 revealed 389 participants without self-reported depression (77.8%) and 94 with self-reported mild depression (18.8%). Self-reported depression differed significantly by discipline, with a significantly higher proportion of physicians reporting depression than nurses (χ^2^ = 12.420, *p* = 0.002: Table 1).

**Table 1:** Survey results for self-reported depression (*N* = 500).

		No		Yes			
	*N*	*n*	%	n	%	χ^2^	*P*
Age							
≤30	246	195	79.3	51	20.7	0.605	0.739
31–40	195	149	76.4	46	23.6		
≥41	59	45	76.3	14	23.7		
Sex							
Male	123	91	74.0	32	26.0	1.376	0.241
Female	377	298	79.0	79	21.0		
Discipline							
Physician	141	95	67.4	46	32.6	12.420	0.002
Nurse	313	257	82.1^a^	56	17.9^a^		
Other medical personnel, technicians, etc.	46	37	80.4	9	19.6		
Level of professional title							
Primary	346	275	79.5	71	20.5	4.442	0.108
Intermediate	136	98	72.1	38	27.9		
Advanced	18	16	88.9	2	11.1		

^a^Compared with the first group, *p* < 0.017.

Univariate logistic regression analysis revealed that the risk of depression was lower in nurses than in physicians (OR = 0.45, 95% CI: 0.29–0.71). Multivariate logistic regression analysis and factor screening showed that the risk of depression among nursing staff was 0.45 times that of physicians (OR = 0.45, 95% CI: 0.29–0.71; Table 2).

**Table 2:** Multivariate logistic regression analysis of factors associated with depression.

						95% CI	
	B	SE	Wald	*P*	OR	Lower limit	Upper limit
Discipline (Reference group: Physician)							
Nurse	−0.798	0.232	11.804	0.001	0.45	0.29	0.71
Other medical personnel, technicians, etc.	−0.688	0.413	2.781	0.095	0.50	0.22	1.13
Constant	−0.725	0.180	16.301	<0.001	0.48		

In the multivariate logistic regression analysis, the independent variables included in the equation were age of the medical personnel (≤30 years, 31–40 years, ≥41 years), sex (male, female), discipline (physician; nurse; other medical personnel, technicians, etc.; medical personnel in administrative logistics), level of professional title (primary, intermediate, senior), and worksite (shelter, monitoring point, negative pressure ward, isolation hotels, support for external area control). The variables were screened using forward stepwise logistic regression method, P_in_ = 0.05, P_out_ = 0.10.

### 3.2. Prevalence of self-reported anxiety

The results of the GAD-7 revealed 424 participants without self-reported anxiety (84.8%) and 64 with self-reported mild anxiety (12.8%). Anxiety also was reported by a significantly higher proportion of men than women (χ^2^ = 5.768, *p* = 0.016) and differed by discipline, with a significantly higher proportion of physicians reporting anxiety than nurses (χ^2^ = 17.862, *p* < 0.001; Table 3).

**Table 3:** Survey results for self-reported anxiety (*N* = 500).

		No		Yes			
	*N*	*n*	%	n	%	χ^2^	*P*
Age							
≤30	246	215	87.4	31	12.6	2.572	0.276
31–40	195	160	82.1	35	17.9		
≥41	59	49	83.1	10	16.9		
Sex							
Male	123	96	78.0	27	22.0	5.768	0.016
Female	377	328	87.0	49	13.0		
Discipline							
Physician	141	105	74.5	36	25.5	17.862	<0.001
Nurse	313	281	89.8^a^	32	10.2^a^		
Other medical personnel, technicians, etc.	46	38	82.6	8	17.4		
Level of professional title							
Primary	346	300	86.7	46	13.3	6.230	0.044
Intermediate	136	107	78.7	29	21.3		
Advanced	18	17	94.4	1	5.6		

^a^Compared with the first group, *P* < 0.017.

Univariate logistic regression analysis revealed that women (OR = 0.53, 95% CI: 0.32–0.90) and nursing staff (OR = 0.33, 95% CI: 0.20–0.56) were protective factors for anxiety, and intermediate professional title (OR = 1.77, 95% CI: 1.06–2.96) were risk factors for anxiety. Multivariate logistic regression analysis and factor screening showed that the risk of anxiety among nursing staff was 0.33 times that of physicians (OR = 0.33, 95% CI: 0.20–0.56; Table 4).

**Table 4:** Multivariate logistic regression analysis of factors associated with anxiety.

						95% CI	
	B	SE	Wald	P	OR	Lower limit	Upper limit
Discipline (Reference group: Physician)							
Nurse	−1.102	0.269	16.846	<0.001	0.33	0.20	0.56
Other medical personnel, technicians, etc.	−0.488	0.434	1.261	0.261	0.61	0.26	1.44
Constant	−1.070	0.193	30.718	<0.001	0.34		

Multivariate logistic regression analysis was conducted as described in Table 2.

### 3.3. Prevalence of insomnia symptoms

According to the ISI results, 451 participants did not self-report clinically significant insomnia (90.2%) and 41 self-reported subthreshold insomnia (8.2%). The proportion of insomnia symptoms reported differed according to age group, with significantly more symptoms reported by the group over 41 years of age compared with the group under 30 years (χ^2^ = 8.613, *p* = 0.013; Table 5). The proportion of insomnia symptoms also differed according to level of professional title, with intermediate level staff reporting more serious symptoms than primary staff (χ^2^ = 13.399, *p* = 0.001; Table 5).

**Table 5:** Survey results for self-reported insomnia (*N* = 500).

		No		Yes			
	*N*	*n*	%	*n*	%	χ^2^	*P*
Age							
≤30	246	231	93.9	15	6.1	8.613	0.013
31–40	195	171	87.7	24	12.3		
≥41	59	49	83.1^a^	10	16.9^a^		
Sex							
Male		112	91.1	11	8.9	0.136	0.713
Female		339	89.9	38	10.1		
Discipline							
Physician	141	121	85.8	20	14.2	4.415	0.110
Nurse	313	287	91.7	26	8.3		
Other medical personnel, technicians, etc.	46	43	93.5	3	6.5		
Level of professional title							
Primary	346	323	93.4	23	6.6	13.399	0.001
Intermediate	136	112	82.4^a^	24	17.6^a^		
Advanced	18	16	88.9	2	11.1		

^a^Compared with the first group, *P* < 0.017.

Univariate logistic regression analysis revealed that age (31–40 years OR = 2.16, 95% CI: 1.10–4.24; ≥41 years OR = 3.14, 95% CI: 1.33–7.41) and intermediate professional title (OR = 3.01, 95% CI: 1.63–5.54) were risk factors for insomnia. Multivariate logistic regression analysis and factor screening showed that the risk of insomnia among personnel with intermediate professional titles was 3.01 times higher than that of personnel with primary professional titles (OR = 3.01, 95% CI: 1.63–5.54; Table 6).

**Table 6:** Multivariate logistic regression analysis of factors associated with insomnia.

						95% CI	
	B	SE	Wald	*P*	OR	Lower limit	Upper limit
Level of professional title (Reference group: Primary)							
Intermediate	1.102	0.312	12.491	<0.001	3.01	1.63	5.54
Advanced	0.563	0.780	0.520	0.471	1.76	0.38	8.10
Constant	−2.642	0.216	149.89	<0.001	0.07		

Multivariate logistic regression analysis was conducted as described in Table 2.

## 4. Discussion

Responses to psychological stress include emotional reactions such as anxiety, depression, hypochondriasis, somatization, and compulsive behavior (11). Reactions differ according to stressor, environment, personality, and other factors. Research has shown that the incidence of anxiety and depression symptoms among medical staff was relatively high during the outbreak period (12). In practical work, we should accumulate real-world evidence about the psychological stress response of health care workers to working in a closed-loop management system and implement appropriate response measures accordingly.

The results of the survey showed that the incidence of depression and anxiety was higher in doctors compared with nurses. Medical staff under the age of 30 years had fewer symptoms of insomnia compared with those over the age of 41 years, and the symptoms were more severe in senior medical personnel than in junior medical personnel. The reasons for this were that nurses interacted directly with patients more frequently, spent more time treating patients, and received more frequent opportunities for daily operation training or nursing training. Meanwhile, nurses more frequently engaged in closed-loop management work, so they were generally more familiar with the working process and protective measures that had to be followed in the isolation area. Accordingly, the nursing staff in the closed-loop work environment had a higher level of adaptability to the stresses of the closed-loop work environment. Among the 500 medical personnel surveyed in this study, 49.2% were under the age of 30 years and 69.2% were junior medical personnel. People with mild infection or those who were asymptomatic were isolated in closed-loop management places such as hotels and makeshift hospitals. The number of isolated people was quite large, as was the workload and physical demands of the job, so young medical workers, who generally have greater physical strength, were considered more suitable. In addition, the younger medical workers had had more opportunities to receive prevention training over the preceding three years. Accordingly, the junior medical workers were better at dealing with the stress of the job.

In the face of large-scale public health events such as outbreaks of infectious diseases, front-line medical staff take on greater responsibilities and are at increased risk of mental exhaustion. Therefore, providing support to medical personnel that help them manage their stress is a critical factor in facilitating epidemic prevention and control (13, 14).

To date, most studies on the effects of the COVID-19 pandemic on the mental health of health care workers and patients have focused on conventional hospital environments (15, 16), while other research has compared mental health outcomes between medical and non-medical staff (17). However, there were few studies on the psychological state of medical staff working in closed-loop management environments. Thus, the present findings provide objective data for subsequent research on psychological interventions and should contribute to improving the mental health of medical workers in closed-loop management environments.

On December 26, 2022, the National Health Commission issued a notice regarding the implementation of the “Class B and Class B management” plan for COVID-19. On January 8, 2023, COVID-19 infection was adjusted to “Class B and Class B management,” and the requirement for nucleic acid testing and centralized isolation of all personnel after entering the closed-loop work environment was rescinded. This marked a new stage in prevention and control, and the closed-loop work system has been abolished. Therefore, although closed-loop work is no longer a source of work stress, medical treatment activities, nursing care, and other work are. In this new stage, it will be important for medical institutions to pay attention to the stress levels of medical staff over time, consider how to help them manage their stress and anxiety and to support them in providing care. However, in the future, it will still be necessary for us to implement timely and effective preventive measures based on the results of this study in order to prevent and treat the psychological trauma caused by closed-loop management of medical personnel.

A clear organizational structure should be established. It is important to create a clear organizational structure where personnel clearly understand their roles and responsibilities. At our hospital, the Medical Department is responsible for organizing work meetings on prevention and control during an epidemic, conducting pre-job training and assessing prevention and control knowledge, and managing the workflow of staff in conjunction with administrative departments. All staff are required to sign a letter of commitment before participating in closed-loop work. The Hospital Infection Management Department creates work systems, procedures, and emergency plans related to hospital infection in isolation environments. In addition, the department guides personnel in the implementation of standard prevention measures, develops scientific protection systems and guidelines for personnel in different positions and with different exposure risks, and is responsible for pre-job training and assessing staff. The Nursing Department is responsible for assigning nursing staff to the isolation working groups and assists the Medical Department with prevention and control training for the support reserve team. The Human Resources Department reviews the qualifications of the health professional technicians selected by each department to work in the closed-loop work environments, conducts psychological evaluations of the staff before they enter these environments, and completes a follow-up evaluation in the medium term. Through multi-department cooperation, we can jointly build a good medical humanistic care service environment. The medical department, hospital infection management department, nursing department and other departments shall ensure the safe working conditions of medical personnel. In addition, the department handles payroll and personnel matters, prioritizes the promotion of relevant personnel, and implements care measures for medical staff with the hospital labor union and the Ministry of Social Work in order to ensure the physical and mental health of medical staff. The department of human resources and the department of education continue to promote medical humanistic education and build a harmonious hospital.

Training should be standardized, with a focus on protection. All personnel in a closed-loop working environment should receive training on how best to protect the hospital, and this should be organized carefully. Personnel at shelter hospitals should also receive skills training covering secondary protection, medical waste treatment, and personal first-aid treatment under secondary protection measures. At our hospital, a total of 540 people participated in such training. Prevention and control training should be provided to medical personnel to ensure that they will be familiar with and can use the relevant isolation technology.

Psychological interventions should be provided and humanistic care measures should be implemented. According to previous research (18), the psychological health of frontline medical staff should be taken into account when formulating relevant measures based on Maslow’s hierarchy of needs theory. In accordance with the guidance on establishing a long-term mechanism for protecting and providing care for medical personnel (19) as well as other notification documents, hospitals should pay attention to the safety and welfare of medical personnel involved in prevention and control work and also work to strengthen psychological care for them (20). During the 3 years of the COVID-19 pandemic, our hospital invited national second-level psychological counselors to give online lectures on mental health to our staff. The personnel at the isolation hotels were provided with sports and music equipment (21–23), which improved their satisfaction and alleviated their stress through participation in sports and music therapy. It is suggested that the facility’s psychological support working group provide psychological interventions when necessary. Management at our hospital ensured the necessary comfort and well-being of personnel at work, by organizing rich cultural and recreational activities among hospital staff, including photography and painting competitions with anti-pandemic themes, and leading by example and reminding personnel of their original intention (24). These measures greatly encouraged the hospital staff to maintain their response readiness.

With the release of the “20 Articles” and “10 New Articles” of the prevention and control policy, medical institutions are facing greater challenges. In the new stage of epidemic prevention and control since December 2022, our hospital has actively responded to the situation and dispatched additional doctors to support the fever clinic as well as performed risk screening, disease diagnosis, and critical identification quickly and accurately. In addition, we have provided pre-hospital emergency treatment, managed the treatment of a large number of critical patients, and strengthened our treatment prioritization procedures and diversity of treatments available. We have implemented pre-job training and assessment for emergency rescue team members and formed an emergency rescue team for severe COVID-19 infection, thereby ensuring emergency rescue and normal medical operations. We also strengthened the security of working conditions, adjusted the protection level, and implemented personal protective measures for medical personnel. We dynamically adjusted the allocation of personnel in all departments and groups in the hospital in order to meet the demand for clinical diagnosis and treatment by combining admission and integrating resources. We also provided medical services online, including consultations and re-consultations, making it easier for people to seek medical care while maximizing the efficiency of medical resources. We strive to arrange the medical staff’s days off to prevent an increase in occupational exposure risk due to decline in physical fitness.

It is important to implement policies to incentivize staff and ensure their safety, especially for those working in front-line prevention and control. Work performance in prevention and control is an important factor in promotions and the awarding of professional titles, and workers recognized for their outstanding performance will be given preferential consideration. It is important to analyze the situation carefully and distribute temporary work subsidies accordingly and as needed. Also, incentivization policies are important such as performance pay for prevention and control personnel. Close attention should be paid to the mental health status of medical staff and additional care should be provided as necessary in order to reduce physical and mental fatigue (25).

At present, protecting health and preventing severe illness is a major challenge for medical institutions at all levels. Many governments have issued policies that incentivize the provision of care by medical personnel, require improvements to their working conditions (26), encourage reasonable shift arrangements, ensure reasonable rest and vacation time for front-line medical workers, and distribute subsidies to medical personnel directly involved in front-line prevention and treatment during epidemics. Going forward, we should work to establish a complete salary and welfare system that considers the clinical practice environment and social status of personnel. In addition, hospitals should provide care and support to third-party personnel such as security, cleaning, distribution, administrative, and other staff in order to ensure normal daily medical operations.

### 4.1. Limitation of this study

Firstly, the survey was conducted during pre-job training only before entering the closed-loop work environment and therefore there was no comparison with working in the closed-loop work environment. In the future, following personnels’ mental health status from before to after entering a closed-loop work environment, as well as making a comparative analysis or a combined analysis involving a psychological survey data of medical staff and/or guests in centralized areas under isolation, would provide more meaningful statistical results and would better guide the implementation of additional psychological intervention measures.

Secondly, the questionnaire did not assess the respondents’ sources of stress, which were considered to be mainly high work-related risks and fear of infection. When the pressure and intensity of work are high, it is easy for workers to burn out. This study lacked the research on the influence factors of external environment on the stress of medical staff. Good financial conditions, strong family support, public recognition and respect are often considered protective factors for anxiety, depression and insomnia. Good economic conditions are a safeguard measure and sense of security, so the inner shock may be less in the face of emergencies. The medical staff will be fully engaged in epidemic control work and actively face the negative effects with the stronger the family support or more respect of the public during closed loop.

Thirdly, the specific work positions and division of labor of medical personnel involved in the prevention and control of epidemics, including direct contact with patients in high-risk positions and coordination work in low-risk positions, may lead to different psychological reactions due to the nature of the work. However, this study did not consider the differences in risks according to position, and this is left to future studies to examine (27).

Finally, with the cancelation of post-entry nucleic acid testing and centralized quarantine for all personnel in China, the closed-loop management system has been completely abolished. This study only conducted a cluster sampling survey of Guangzhou Panyu Central Hospital, and thus there were no sampling surveys or comparative analyses with other medical institutions. This study will continue in the future. Cluster sampling surveys also will be carried out with other medical institutions when closed-loop management work maybe again implemented in response to a public health emergency.

## 5. Conclusion

In summary, during the COVID-19 pandemic, conducting psychological health assessments during pre-job training for medical personnel participating in closed-loop management by medical institutions was beneficial for the timely detection of psychological problems. In future outbreaks of infectious diseases, it will be necessary to strengthen early psychological counseling and interventions, especially for senior male physicians working in closed-loop management environments.

## Data availability statement

The original contributions presented in the study are included in the article/supplementary material, further inquiries can be directed to the corresponding authors.

## Ethics statement

The studies involving humans were approved by the Guangzhou Panyu Central Hospital. The studies were conducted in accordance with the local legislation and institutional requirements. Written informed consent for participation in this study was provided by the participants’ legal guardians/next of kin.

## Author contributions

LZ: Conceptualization, Data curation, Writing – original draft. LH: Data curation, Writing – original draft. JH: Formal analysis, Writing – original draft. JQ: Investigation, Writing – original draft. ZF: Conceptualization, Writing – original draft. JC: Writing – review & editing. XH: Writing – review & editing. CH: Writing – review & editing.
